# Modeling and Optimizing a New Culture Medium for *In Vitro* Rooting of G×N15 *Prunus* Rootstock using Artificial Neural Network-Genetic Algorithm

**DOI:** 10.1038/s41598-018-27858-4

**Published:** 2018-07-02

**Authors:** Mohammad Mehdi Arab, Abbas Yadollahi, Maliheh Eftekhari, Hamed Ahmadi, Mohammad Akbari, Saadat Sarikhani Khorami

**Affiliations:** 1 Department of Horticultural Science, Faculty of Agriculture, Tarbiat Modares University (TMU), Tehran, Iran; 2 Department of Horticulture, College of Aburaihan, University of Tehran (UT), Tehran, Iran; 3 Bioscience and Agriculture Modeling Research Unit, College of Agriculture, Tarbiat Modares University, Tehran, Iran; 4 Department of Horticultural Sciences, Faculty of Agriculture, University of Tabriz, Tabriz, Iran

## Abstract

The main aim of the present investigation is modeling and optimization of a new culture medium for *in vitro* rooting of G×N15 rootstock using an artificial neural network-genetic algorithm (ANN-GA). Six experiments for assessing different media culture, various concentrations of Indole – 3- butyric acid, different concentrations of Thiamine and Fe-EDDHA were designed. The effects of five ionic macronutrients (NH_4_^+^, NO_3_^−^, Ca^2+^, K^+^ and Cl^−^) on five growth parameters [root number (RN), root length (RL), root percentage (R%), fresh (FW) and dry weight (DW)] were evaluated using the ANN-GA method. The R^2^ correlation values of 0.88, 0.88, 0.98, 0.94 and 0.87 between observed and predicted values were acquired for all five growth parameters, respectively. The ANN-GA results indicated that among the input variables, K^+^ (7.6) and NH4^+^ (4.4), K^+^ (7.7) and Ca^2+^ (2.8), K^+^ (36.7) and NH_4_^+^ (4.3), K^+^ (14.7) and NH_4_^+^ (4.4) and K^+^ (7.6) and NH_4_^+^ (4.3) had the highest values of variable sensitivity ratio (VSR) in the data set, for RN, RL, R%, FW and DW, respectively. ANN-GA optimized LS medium for G×N15 rooting contained optimized amounts of 1 mg L^−1^ IBA, 100, 150, or 200 mg L^−1^ Fe-EDDHA and 1.6 mg L^−1^ Thiamine. The efficiency of the optimized culture media was compared to other standard media for *Prunus* rooting and the results indicated that the optimized medium is more efficient than the others.

## Introduction

In recent decades, interspecies hybrids of the *Prunus* genus have been widely used as a rootstock in developing countries, which has solved major problems in stone fruit trees^1^. Garnem (G×N15) is one of its improved outcomes, which is a hybrid between the almond and peach [*Prunus amygdalus* (Garfi) × *Prunus persica* (Nemared)] that developed at the Center of Investigation and Technology Agrifood of Aragon in Spain^2,3^. Garnem is a strong, early bearing rootstock compatible with all types of soils with good drainage, tolerant to salinity, drought, waterlogging, iron chlorosis, nematodes, and soil-borne diseases which are suitable for both irrigated and non-irrigated areas^2,4–6^. In order to create mechanized orchards, its propagation via tissue culture is essential. However, rooting is a hard step in tissue culture propagation of many fruit trees such as *Prunus* rootstocks and varieties^7^.

Different physiological, biochemical, and genetic factors such as genotype/cultivars, medium composition, plant growth regulators, and also physical factors affect rooting^8^. Diverse culture media have different effects on rooting stage because of their different nutrient concentrations, so applying a specific medium depends on the plant species^9^. To improve the *in vitro* rooting in most plants, the macro elements have been reduced from the medium and also various vitamin combinations have been applied^10,11^. Exogenously added auxins such as indole-3-butyric acid (IBA), 1-naphthalene acetic acid (NAA), and indole-3-acetic acid (IAA), are able to induce adventitious roots. However, this requirement is only at early stages of the rooting process for promoting the adventitious roots^12,13^. In the next stages of root development, it can change or even inhibit the development of the rooting system^14^. Depending on the plant species, different kind of auxins might be effective on rooting^11,15^. The composition of root initiation medium such as hormonal content or the presence of active charcoal could influence the rooting process. When the medium contained active charcoal, the effect of NAA was reduced probably because of the adsorption of additional NAA by active charcoal^11^.

The hormonal composition of the last proliferation medium could influence the rooting ability of *in vitro* micro-shoots^10^. Although the high level of IBA can inhibit the rooting, but when a higher concentration of IBA (0.3 mg L^−1^) was applied in combination of different cytokinins in the last proliferation medium, it tended to enhance the root number independent of IBA concentration^16^. It has been expressed that phloroglucinol would increase the rooting due to its auxin activity and ½ MS medium supplemented with 0.2 mg L^−1^ IBA and 40 mg L^−1^ phloroglucinol have had the maximum rooting^8^. In a micropropagation study of *Prunus domestica* using ½ MS supplemented with 1 mg L^−1^ NAA, 0.1 mg L^−1^ GA3 and 20 g sucrose, 85% rooting was reported^17^. Also, different sources of iron have various effects on *in vitro* rooting of fruit trees. Substituting the iron source from Fe-EDDHA to FeCl_3_ improved the *in vitro* rooting of GF677 rootstocks, and increased the root fresh weight and dry weight^18^. In a study that investigated the effect of Fe-EDDHA and ascorbic acid on *in vitro* rooting of GF677 rootstock, the best rooting results were observed after four weeks of culture with 280 mg L^−1^ Fe-EDDHA. They also reported that ascorbic acid had no distinct stimulating effect on rooting^19^.

Although there are several biological processes that can be easily observed in plant tissue culture, none of them are linear and also would be affected by numerous other factors; therefore, the appropriate modeling can effectively predict the *in vitro* growth kinetics, and the plant biomass^20,21^. Conventional analytical techniques based on mathematical models are questionable for these purposes because these methods do not conform to the non-idealities of *in vitro* culture process^22^. Our previous report indicate that artificial neural network-genetic algorithm (ANN-GA) is better than traditional regression methods such as forward, backward or stepwise to predict and optimize new culture media^23^. Artificial neural networks (ANN) are inspired by the human brain functions^24^. Although ANN has shown significant progress in biological processes, they have rarely been used in complicated plant tissue culture systems. ANN based modeling methods are more flexible and useful in dealing with non-linear relationships existing in tissue culture practices. ANN doesn’t need any previous knowledge concerning the construction or interrelationships between input and output signals which is one of its benefits^22^. Other advantages of ANN are predicting the plant biomass^25^ and clustering the *in vitro* regenerated plantlets. Also, affecting the growth and quality of regenerated plants by controlling CO_2_, light, ventilation, and air temperature inside the culture vessels could be expressed as ANN advantages^22^.

**Figure 1:**
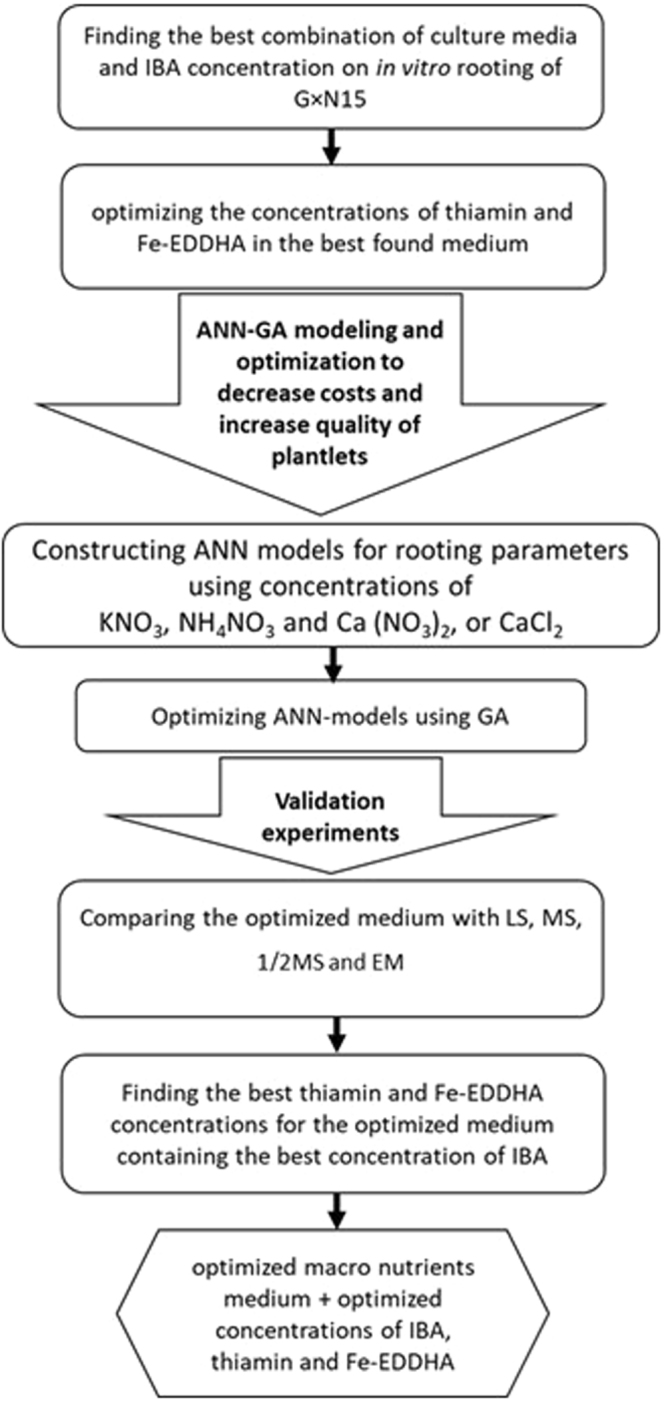
Overall schematic of stages to achieve an optimized culture medium for *in vitro* rooting of G×N15 *prunus* rootstock.

Understanding the relationship between the culture conditions and plant growth parameters is a foundation towards the development of high quality *in vitro* plantlets. In a modeling study for direct rooting and acclimatization of grape (*Vitis vinifera* L.) by neurofuzzy logic approach, the input data were three variables including types of auxin, auxin concentration and sucrose concentration, and the output data were root length and root number^26^. In several studies of micropropagation using bioreactors, input data such as pH, volume of growth medium, sucrose content, nitrate concentration, temperature, time of inoculation, size, fresh weight, and number of explant per flask in output of root weight and biomass using neural networks for modeling and optimization of rooting have been reported^27–29^. In our previous works, we found ANN-GA as a very precise and powerful modeling technique to optimizing nutrients for pear rootstocks^30^ and nutrients and hormonal composition for G×N 15 *Prunus* rootstock^20,23^. Recently, this technique has been also used successfully for predicting and optimizing the effect of various media components on *Pistacia vera* proliferation^31^. But there is lack of information concerning the effectiveness of this efficient method (ANN-GA) to find the complicated and non-linear relationships among important culture medium components and rooting. Establishing an efficient root structure on shoots grown *in vitro* is essential for massive commercial micropropagation purposes and plays a vital role in acclimatization of *in vitro* plantlets. As the most plantlet mortality occur during this phase and imposes large economical losses to the plant producers. So, we did this research distinctly on rooting to find the best composition of nutrients in general and in combination with thiamin vitamin and the chelated form of iron i.e. ethylene diamine di-o-hydroxyphenyl acetic acid (Fe-EDDHA) (6% Fe) as well as rooting hormone IBA which are well-known as effective factors in producing large number of high quality *in vitro* roots^18^. Furthermore, the present comprehensive study about effective factors on *in vitro* rooting using ANN-GA modeling and optimization method was followed with practical testing of the optimized medium which could confirm our theoretical results (Fig. 1).

Considering the above mentioned studies^20,23,30,31^ on proliferation stage of micropropagation process, the hypothesis of the present investigation is that different compositions of culture media supplemented with different concentrations of three important factors affecting on rooting including IBA, Thiamine and Fe-EDDHA determine the *in vitro* rooting efficiency. So, the ultimate goal of the current study is to develop a precise ANN-GA model for efficient and reproducible protocol by achieving an optimal nutrient culture medium supplemented with optimal concentrations of IBA, Thiamine and Fe-EDDHA concentrations.

## Results

According to the analysis of variance for the first experiments, the interaction of culture medium and hormone significantly affects the rooting factors such as the root number (RN), root length (RL), root percentage (R%), and root fresh weight (FW) and dry weight (DW). The maximum RN with an average of 13.2 (the number of new roots per explant) in LS culture medium containing 1 mg L^−1^ IBA and the minimum RN in EM culture medium containing 0.5 mg L^−1^ of IBA was observed (Table 1). Maximum R% with an average of 80% in LS culture medium containing 1 mg L^−1^ IBA and the minimum R% in the MS culture medium containing 2 and 1.5 mg L^−1^ of IBA was observed. The highest FW with an average of 0.59 g was observed in LS culture medium containing 1 mg L^−1^ IBA (Table 1).

**Table 1:** Effect of different culture media and concentration of IBA on Root Number (RN), Root length (RL), root percentage (R%), fresh weight (FW) and dry weight (DW).

Effects		RN	RL	R%	FW	DW
**Medium × IBA (mg L** ^**−1**^ **)**						
MS	0	0.0 ± 0.00 h	0.0 ± 0.00 g	0.0 ± 0.00 g	0.0 ± 0.00 k	0.0 ± 0.00 e
	0.05	3.4 ± 0.24 ef	5.30 ± 0.18 cd	0.25 ± 0.00 def	0.16 ± 0.01 fg	0.014 ± 0.002 de
	1	5.2 ± 0.20 d	5.68 ± 0.22 bc	0.30 ± 0.05 cdef	0.21 ± 0.006 f	0.030 ± 0.003 cde
	1.5	3.2 ± 0.20 ef	4.52 ± 0.18 ef	0.10 ± 0.06 fg	0.14 ± 0.005 gh	0.018 ± 0.002 cde
	2	1.8 ± 0.20 g	3.92 ± 0.19 f	0.10 ± 0.06 fg	0.07 ± 0.001 ij	0.041 ± 0.012 cd
EM	0	0.0 ± 0.00 h	0.0 ± 0.00 g	0.0 ± 0.00 g	0.0 ± 0.00 k	0.0 ± 0.00 e
	0.05	2.2 ± 0.20 fg	3.86 ± 0.11 f	0.25 ± 0.08 def	0.05 ± 0.006 jk	0.037 ± 0.011 cd
	1	3.6 ± 0.24 e	4.40 ± 0.08 ef	0.35 ± 0.06 cde	0.09 ± 0.005 ij	0.017 ± 0.001 cde
	1.5	3.2 ± 0.37 ef	4.20 ± 0.18 ef	0.35 ± 0.06 cde	0.11 ± 0.005 hi	0.027 ± 0.013 cde
	2	4.4 ± 0.24 de	5.26 ± 0.17 cd	0.20 ± 0.09 efg	0.16 ± 0.011 fg	0.018 ± 0.002 cde
½ MS	0	0.0 ± 0.00 h	0.0 ± 0.00 g	0.0 ± 0.00 g	0.0 ± 0.00 k	0.0 ± 0.00 e
	0.05	7.0 ± 0.32 c	5.74 ± 0.06 bc	0.45 ± 0.05 cd	0.32 ± 0.009 d	0.048 ± 0.004 bc
	1	9.0 ± 0.32 b	6.36 ± 0.17 b	0.75 ± 0.00 a	0.44 ± 0.010 b	0.079 ± 0.003 b
	1.5	5.4 ± 0.24 d	5.46 ± 0.09 cd	0.50 ± 0.05 bc	0.26 ± 0.002 e	0.041 ± 0.001 cd
	2	3.2 ± 0.20 ef	4.86 ± 0.07 de	0.25 ± 0.00 def	0.17 ± 0.017 fg	0.038 ± 0.013 cd
LS	0	0.0 ± 0.00 h	0.0 ± 0.00 g	0.0 ± 0.00 g	0.0 ± 0.00 k	0.0 ± 0.00 e
	0.05	7.8 ± 0.20 bc	6.36 ± 0.06 b	0.70 ± 0.05 ab	0.41 ± 0.005 bc	0.075 ± 0.003 b
	1	13.2 ± 0.37 a	7.58 ± 0.25 a	0.80 ± 0.05 a	0.59 ± 0.008 a	0.128 ± 0.008 a
	1.5	7.2 ± 0.37 c	6.34 ± 0.08 b	0.50 ± 0.00 bc	0.37 ± 0.014 c	0.049 ± 0.003 bc
	2	4.2 ± 0.20 de	5.50 ± 0.13 cd	0.25 ± 0.05 def	0.21 ± 0.022 f	0.026 ± 0.005 cde

Values in each column represent means ± SE. Different letters within columns indicate significant differences (p < 0.01).

Results from the second experiment indicate that Thiamine and Fe-EDDHA and also their interactions significantly affect the RN, RL, R% and FW and DW. The maximum RN with an average of 14 (the number of new roots per explant) observed in 150 mg L^−1^ Fe-EDDHA and 1.6 mg L^−1^ Thiamine and the minimum RN with an average of 2.6 obtained in 200 mg L^−1^ Fe-EDDHA and 4 mg L^−1^ Thiamine. Also, the maximum and the minimum rooting with an average of 100% and 25% was obtained from 100 and 150 mg L^−1^ Fe-EDDHA in combination with the 1.6 mg L^−1^ Thiamine and 200 mg L^−1^ Fe-EDDHA in combination with 4 mg L^−1^ Thiamine, subsequently. Treatment containing 150 mg L^−1^ Fe-EDDHA combining with 1.6 mg L^−1^ Thiamine had the maximum fresh root weight with an average of 0.48 g (Table 2).

**Table 2:** Effect of different concentrations of Fe-EDDHA and Thiamine on root number (RN), root length (RL), root percentage (R%), fresh weight (FW) and dry weight (DW).

Effects		RN	RL	R%	FW	DW
**Thiamine × Fe-EDDHA (mg L** ^**−1**^ **)**						
0.4	100	6.4 ± 0.51 de	6.42 ± 0.08 de	0.55 ± 0.05 def	0.39 ± 0.006 bc	0.07 ± 0.003 c
	150	8.4 ± 0.24 c	7.96 ± 0.19 bc	0.75 ± 0.00 bcd	0.45 ± 0.010 a	0.12 ± 0.010 ab
	200	5.6 ± 0.24 e	5.78 ± 0.15 ef	0.50 ± 0.05 ef	0.34 ± 0.002 d	0.06 ± 0.003 c
1.6	100	10.2 ± 0.37 b	8.20 ± 0.10 b	1.00 ± 0.00 a	0.40 ± 0.003 bc	0.10 ± 0.003 b
	150	14.0 ± 0.32 a	9.44 ± 0.46 a	1.00 ± 0.00 a	0.48 ± 0.010 a	0.14 ± 0.006 a
	200	7.6 ± 0.40 cd	7.82 ± 0.09 bc	0.80 ± 0.05 abc	0.35 ± 0.003 cd	0.06 ± 0.002 c
2.8	100	8.2 ± 0.20 c	7.14 ± 0.10 cd	0.75 ± 0.08 bcd	0.36 ± 0.004 bcd	0.06 ± 0.002 c
	150	10.6 ± 0.24 b	8.06 ± 0.16 b	0.95 ± 0.05 ab	0.33 ± 0.010 d	0.04 ± 0.003 de
	200	6.6 ± 0.24 de	7.38 ± 0.18 bc	0.70 ± 0.05 cde	0.33 ± 0.007 d	0.04 ± 0.002 de
4	100	3.2 ± 0.20 f	6.40 ± 0.13 de	0.40 ± 0.06 fg	0.26 ± 0.011 e	0.03 ± 0.002 ef
	150	3.4 ± 0.24 f	5.98 ± 0.07 ef	0.35 ± 0.06 fg	0.26 ± 0.015 e	0.02 ± 0.003 ef
	200	2.6 ± 0.24 f	5.40 ± 0.08 f	0.25 ± 0.00 g	0.16 ± 0.011 f	0.01 ± 0.001 f

Values in each column represent means ± SE. Different letters within columns indicate significant differences (p < 0.01).

### Modeling and evaluation

**Figure 2:**
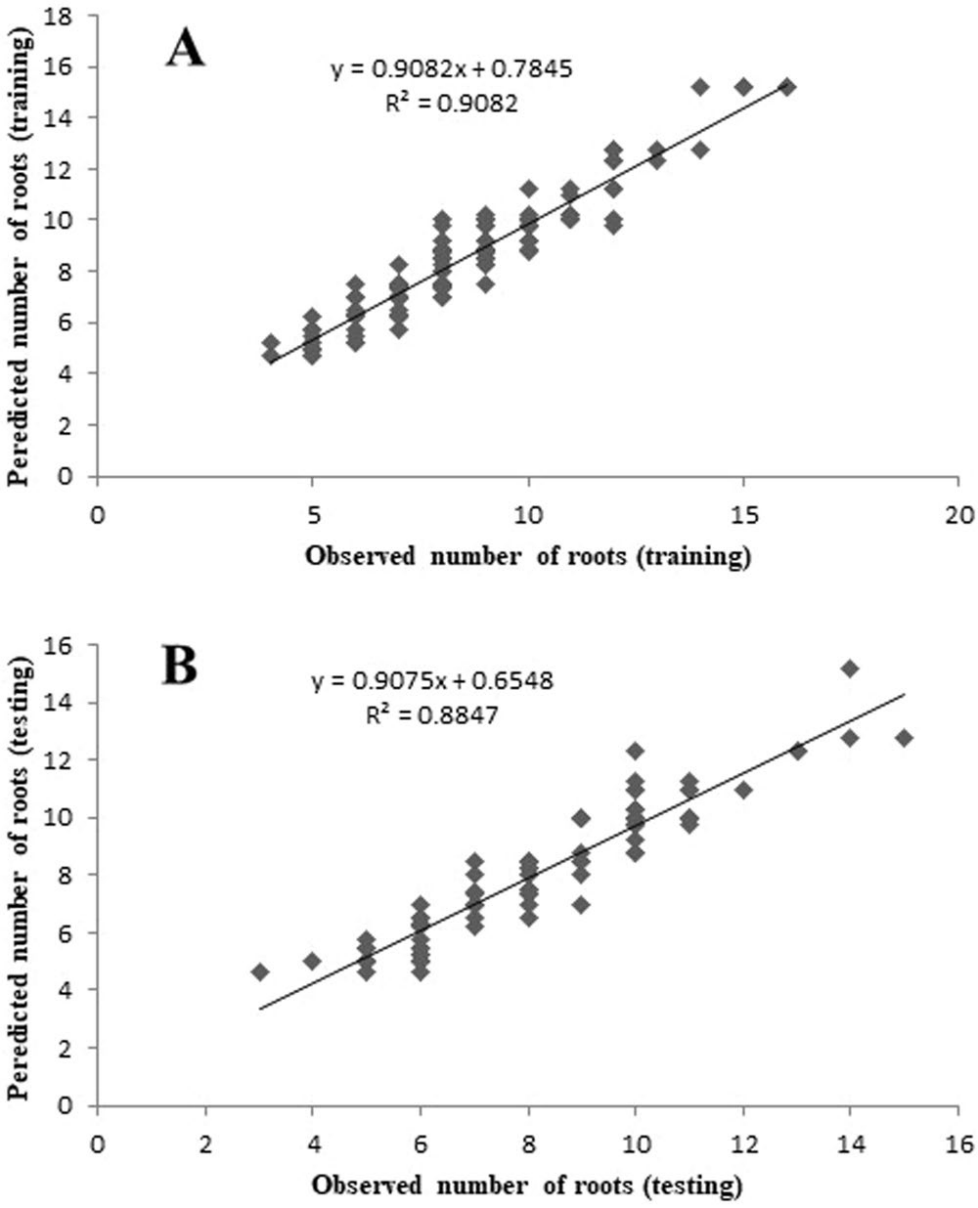
Scatter plot of observed vs. model-predicted values of number of roots of G×N15 rootstock during *in vitro* rooting obtained by artificial neural network model. (**A**) Training set (n = 130); (**B**) testing set (n = 86). The solid line indicates the fitted simple regression line on scatter points.

**Figure 3:**
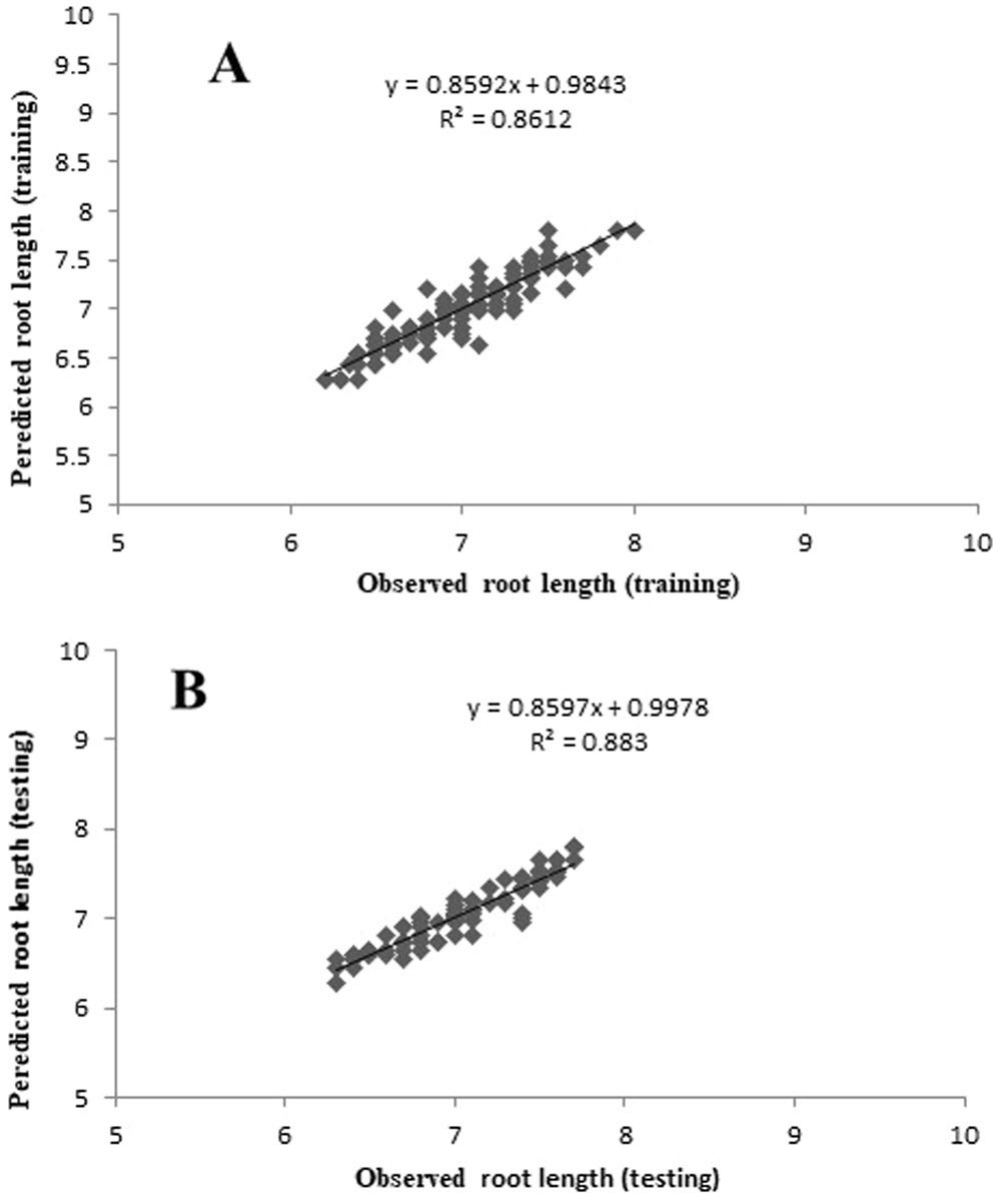
Scatter plot of observed vs. model-predicted values of root length of G×N15 rootstock during *in vitro* rooting obtained by artificial neural network model. (**A**) Training set (n = 130); (**B**) testing set (n = 86). The solid line indicates the fitted simple regression line on scatter points.

**Figure 4:**
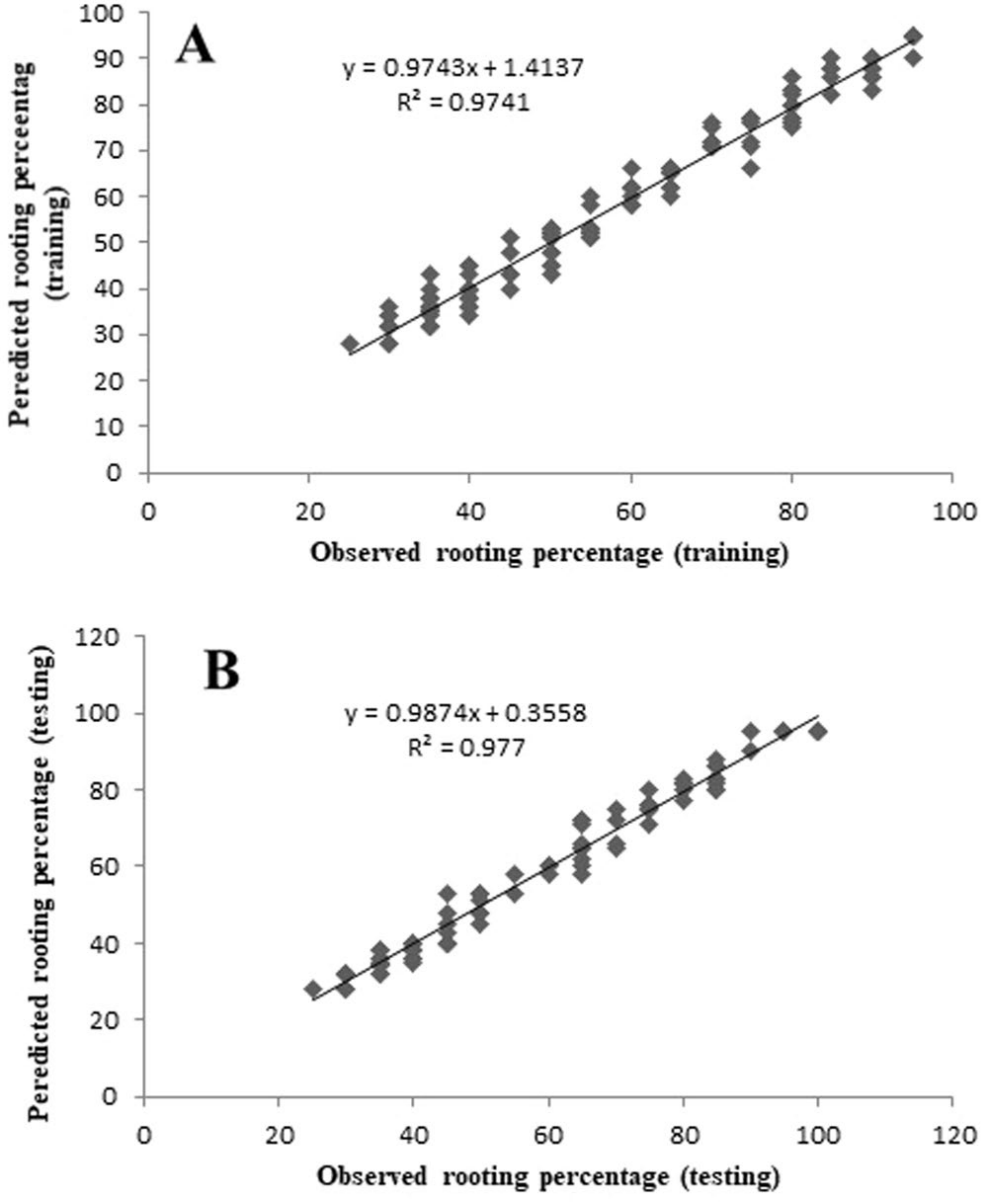
Scatter plot of observed vs. model-predicted values of rooting percentage of G×N15 rootstock during *in vitro* rooting obtained by artificial neural network model. (**A**) Training set (n = 130); (**B**) testing set (n = 86). The solid line indicates the fitted simple regression line on scatter points.

**Figure 5:**
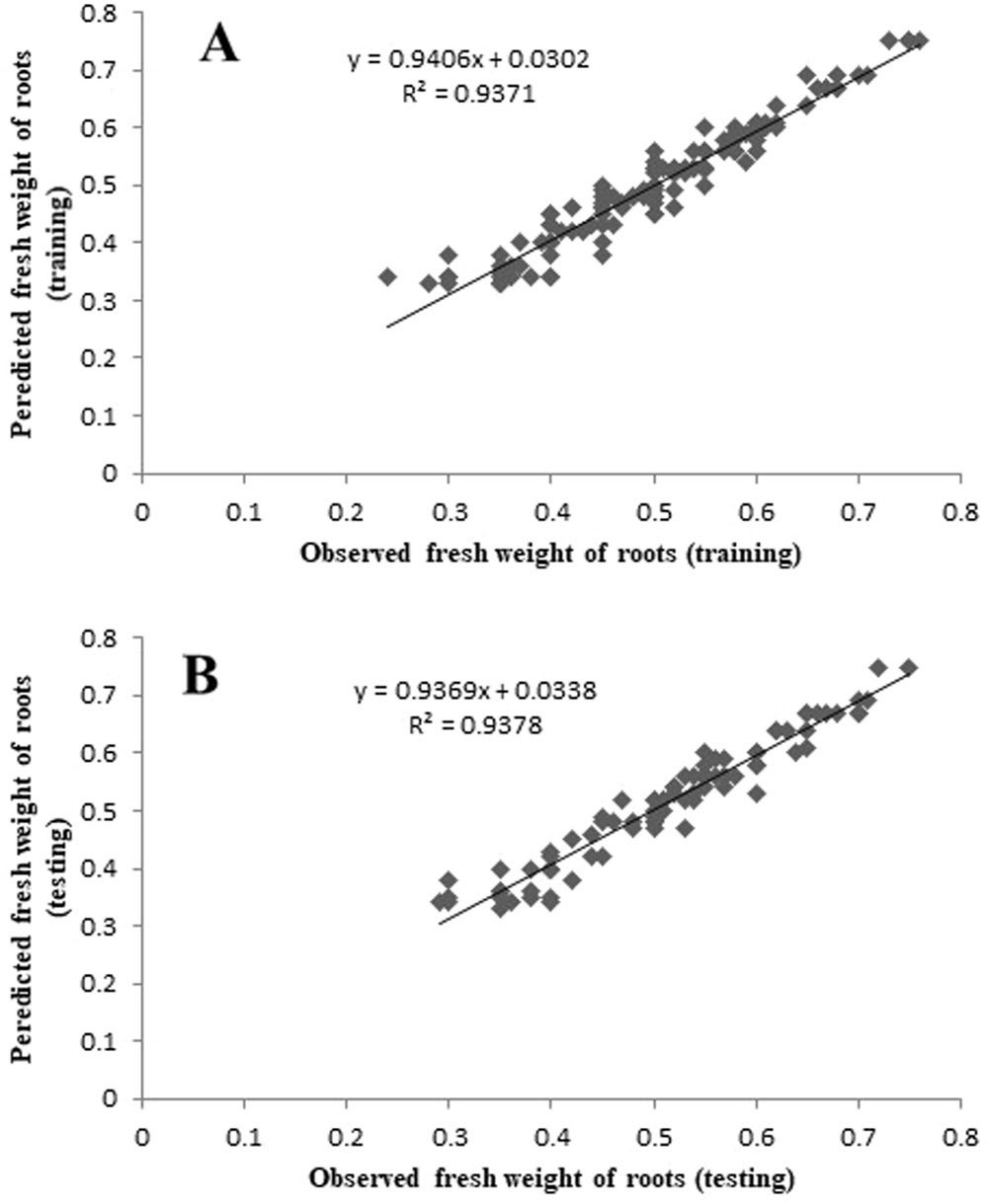
Scatter plot of observed vs. model-predicted values of fresh weight of roots of G×N15 rootstock during *in vitro* rooting obtained by artificial neural network model. (**A**) Training set (n = 130); (**B**) testing set (n = 86). The solid line indicates the fitted simple regression line on scatter points.

**Figure 6:**
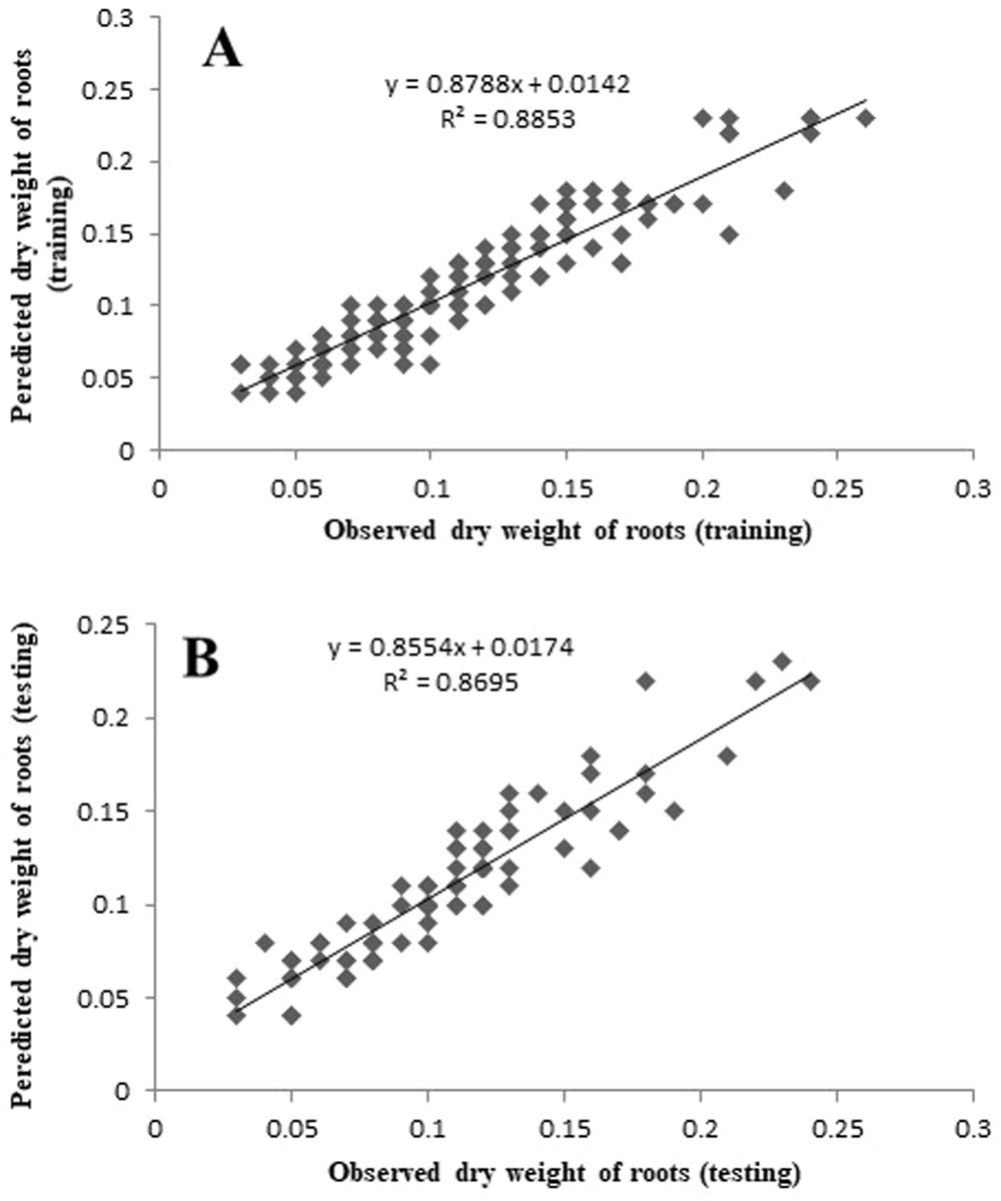
Scatter plot of observed vs. model-predicted values of dry weight of roots of G×N15 rootstock during *in vitro* rooting obtained by artificial neural network model. (**A**) Training set (n = 130); (**B**) testing set (n = 86). The solid line indicates the fitted simple regression line on scatter points.

The plots of ANN model-predicted vs. the observed values of the RN, RL, R%, FW, and DW are shown in Figs 2–6. The fitted simple regression lines indicate good agreement between the observed and predicted values of the RN, RL, R%, FW, and DW for both the training and testing sets. Using high squared determination coefficients (R^2^) fitting method and based on the ANN models obtained, ten graphs were generated to show the variation with RN, RL, R%, FW and DW of NH_4_^+^, NO_3_^−^, Ca^2+^, K^+^, and Cl^−^ (Figs 2–6). The graphs may be useful for understanding the complete nutrient-response relationship and to evaluate the combined effects of LS medium modified mineral nutrients. The goodness-of-fit statistical values derived from the ANN model to predict the RN, RL, R%, FW and DW are shown in Table 3. The ANN models could accurately (R^2^ > 0.88, 0.88, 0.98, 0.94 and 0.87) predict the RN, RL, R%, FW and DW of the testing data sets, which were not used during the training processes (Figs 2–6). Moreover, the trained ANN models of RN, RL, R%, FW and DW had balanced statistical values for the two subsets of training and testing (Table 3). Overall, statistical values (Table 3) revealed that the ANN-based models could efficiently fit data on the responses of G×N15 micro-shoots during *in vitro* rooting to LS medium with modified mineral nutrients.

**Table 3:** Statistics and information on artificial neural network models for root number (RN), root length (RL), root percentage (R%), root fresh weight (FW) and root dry weight (DW) of G×N15 *prunus* rootstock plantlets during *in vitro* rooting (training vs. testing values).

Item	RN		RL		R%		FW		DW	
	Training	Testing	Training	Testing	Training	Testing	Training	Testing	Training	Testing
R Square	0.91	0.88	0.86	0.88	0.97	0.98	0.94	0.94	0.88	0.87
RMSE	0.74	0.84	0.14	0.13	0.032	0.033	0.027	0.027	0.017	0.016
MBE	−0.0003	−0.11	−0.0008	0.012	−0.0008	−0.004	0.0002	0.001	0.0003	0.002

### Sensitivity analysis of the models

The comparative rank of input variables was determined using the entire 216 lines of data (training and testing) to calculate the overall VSR. The VSR obtained for the model output (RN, RL, R%, FW, and DW), with respect to modified mineral nutrients of LS medium are shown in Table 4. Analysis of RN indicated that the number of micro-shoots of G×N15 were more sensitive to K^+^ concentration, followed by NH_4_^+^, Ca^2+^, Cl^−^, and NO_3_^−^ (Table 4). In RL model, the feed efficiency of G×N15 showed more sensitivity to K^+^ concentration, followed by Ca^2+^, NH_4_^+^, Cl^−^, and NO_3_^−^ (Table 4). Analysis of R% indicated that the R% of G×N15 micro-shoots were more sensitive to K^+^ concentration, followed by NH_4_^+^, Cl^−^, Ca^2+^, and NO_3_^−^ (Table 4). In FW, the feed efficiency of G×N15 micro-shoots showed more sensitivity to K^+^ concentration, followed by NH_4_^+^, Cl^−^, Ca^2+^, and NO_3_^−^ (Table 4). Analysis of DW indicated that the DW of G×N15 micro-shoots were more sensitive to K^+^ concentration, followed by NH_4_^+^, Ca^2+^, Cl^−^, and NO_3_^−^ (Table 4). This suggests that ion concentration (inputs) levels can significantly influence the performance of G×N15 micro-shoots rooting. However, the effect of K^+^, NH_4_^+^ and Ca^2+^ levels was more pronounced than that of Cl^−^ and NO_3_^−^. Several researchers have suggested that the responses of *Prunus* rootstocks to K^+^, NH_4_^+^, Ca^2+^, Cl^−^ and NO_3_^−^ are different.

**Table 4:** Importance of ion concentrations (mM) of the different culture media used for *in vitro* rooting of G×N15 rootstock according to the sensitivity analysis on the developed neural network model to rank the importance of ion concentrations.

Element	NO_3_^−^	NH_4_^+^	K^+^	Ca^2+^	Cl^−^
RN	1	4.4	7.6	1.5	1.2
Rank	5	2	1	3	4
RL	1	2.5	7.7	2.8	2
Rank	5	3	1	2	4
R%	1	4.3	36.7	1.2	1.7
Rank	5	2	1	4	3
FW	1	4.4	14.7	2.1	2.9
Rank	5	2	1	4	3
DW	1	4.3	7.6	2	1.4
Rank	5	2	1	3	4

VSR: relative indication of the ratio between the variable sensitivity error and the error of the model when all variables are available.

### Model optimization

The final goal was to analyze the ANN models to address the question of what levels of elements NH_4_^+^, NO_3_^−^, K^+^, Ca^2+^, and Cl^−^ may be used to achieve maximum R%. The purpose of this study, as in many other studies, was not to develop profit (economic) optimization, but to optimize nutrient requirements for the maximum R% of G×N15 micro-shoots. The results of optimization are summarized in Table 5. The process of optimization was conducted in the range of values from the data set (Table 7).

**Table 5:** Optimized artificial neural network models using genetic algorithm to reach maximum root number (RN), root length (RL), root percentage (R%), root fresh weight (FW) and root dry weight (DW) of G×N15 *prunus* rootstock plantlets during *in vitro* rooting.

Item G×**N15**	Input variable [Ion concentrations (mM)]					Predicted output variable at optimal point
	NO_3_^−^	NH_4_^+^	K^+^	Ca^2+^	Cl^−^	
RN	37.7	17.5	19.7	2.9	0.78	15.2
RL	34.8	11	18.2	1.6	2.3	7.8
R%	42.9	18.1	10.3	2.9	1.2	0.95
FW	36.4	20.6	9.4	2.6	1.3	0.069
DW	25	19.4	12.5	1.4	0.07	0.023

In conclusion, a platform of ANN-based models with sensitivity analysis and optimization algorithms was used successfully in this study to integrate published data on the responses of *in vitro* rooting of G×N15 rootstock to macro element nutrient concentration. Analyses of the ANN models for RN, RL, R%, FW and DW from a compiled data set suggested that the K^+^, NH_4_^+^ and Ca^2+^ concentrations were more important than the NO_3_^−^and Cl^−^ concentrations. According to the preliminary results obtained by the ANN-GA, optimal productivity (R%) may be achieved with modified LS medium containing 42.9 mM of NO_3_^−^, 18.1 mM of NH_4_^+^, 10.3 mM of K^+^, 2.9 mM of Ca^2+^ and 1.2 mM of Cl^−^. Finally, modified LS medium was compared with other media, such as MS, EM, ½ MS and LS that are commonly applied for *Prunus in vitro* rooting (Table 10).

### Validation experiment (assessment of optimum productivity of new media formulated)

When MS, EM, ½ MS, LS and modified LS were used, multifactorial analysis of variance showed that different types of media had significant effects (P < 0.001) on RN, RL, R%, FW and DW of G×N15 rootstock after four weeks in culture. The best root formation was obtained on modified LS medium. Modified LS medium including 1 mg L^−1^ IBA resulted in production of an average 15.4 new root per treated explant which is significantly higher than that produced by using the same concentration of IBA in other media. LS and modified LS media were the most productive media and, MS and EM media were the poorest performer for *in vitro* rooting of G×N15 rootstock. The highest average RL (8.10 cm) obtained by modified LS medium was also significantly higher than those with MS, EM, ½ MS and LS media. Modified LS medium supplemented with 1 mg L^−1^ IBA resulted in the highest R% with an average 95%, which was significantly higher than that produced by other media. The highest FW with an average 0.70 g per explant was obtained in modified LS medium supplemented with 1 mg L^−1^ IBA. Based on the above results, it can be inferred that modified LS medium was more efficient than other media.

### The effects of Fe-EDDHA and Thiamine in modified LS medium optimized by ANN-GA on *in-vitro* rooting of G×N15 rootstock

Fe-EDDHA, Thiamine and also their interaction could significantly affect the RN, RL, R%, FW and DW in the modified LS medium. 150 mg L^−1^ Fe-EDDHA and 1.6 mg L^−1^ Thiamine produced the maximum RN with an average of 16 (the RN per explant) whereas, the minimum RN with an average of 3.4 observed in treatments with 200 mg L^−1^ Fe-EDDHA and 4 mg L^−1^ Thiamine (Table 7). Also, the maximum R% with an average of 100% observed in 100, 150, and 200 mg L^−1^ Fe-EDDHA along with 1.6 mg L^−1^ Thiamine and the minimum R% with an average of 25% belonged to 200 mg L^−1^ Fe-EDDHA combined with 4 mg L^−1^ thiamine (Table 7). Also, in the treatments containing 150 mg L^−1^ Fe-EDDHA and 2.8 mg L^−1^ Thiamine, the R% with an average of 100% was observed. Treatments containing 150 mg L^−1^ Fe-EDDHA and 1.6 mg L^−1^ thiamine had the maximum FW with an average of 0.57 g per each rooted micro-shoot.

## Discussion

Micropropagation is a very complicated and time-consuming process which needs expensive chemicals to set a protocol for a plant. Specifically, fruit trees are harder to work with than herbal plants, so that producing tree plantlets such as *Prunus* rootstocks with high quality roots which could prevent plantlet losses during hardening phase is a very difficult step affected by different factors such as genotype, medium composition and plant growth regulators^7,8^. Various culture media have different effects at different stages of plant micropropagation because of their different nutrient concentrations and compositions. On the other hand, each plant species shows a different reaction to a special culture medium, so one has to optimize a unique medium for each unique plant species and each micropropagation phase. The importance of using ANN-GA mathematical modeling method as a very accurate procedure for optimizing culture media in plant tissue culture technique has been recently considered. Different media ingredients like nutrients, hormones and vitamins have been used as inputs of ANN for modeling and predicting their effects on proliferation stage of a few number of tree species like *Pistacia vera*^31^, pear rootstoks^30^ and G×N15 *prunus* rootstock^20,23^. All these researches found ANN-GA as a powerful tool in designing optimized proliferation culture media and predicting the proliferation indices by using different amounts of ingredients. Hence, in the present work, we tried to introduce a particular and optimized culture medium for rooting, as a fatal phase of plant *in vitro* regeneration, using ANN-GA which to our knowledge has not been previously done on any of the woody or herbal plants, although the neurofuzzy logic^26^ and ANN^32^ have been used for modeling *Vitis vinifera in vitro* rooting. Here, we practically tested the optimized medium in contrast to other media (Table 10), so, this work can be an important guide for future studies on *in vitro* rooting of other plant species such as hard-to-root ones.

The plants ability to produce lateral roots relies on the interaction of many external and internal factors such as hormone types and elements of culture media. Exogenously added auxins such as IBA, NAA, and IAA, are able to induce adventitious roots and are just needed in early stages of the rooting process for promoting the adventitious roots^12,13^. In the present investigation, the interaction between medium type and various hormones had different effects on rooting. The maximum R% was observed in LS medium containing 1 mg L^−1^ IBA condition. The results obtained here is in keeping with^9^ which mentioned that ½ MS culture medium is suitable for *in vitro* rooting of *Prunus* rootstock.

Analysis of rooting elements through ANN-GA indicates that potassium nitrate, ammonium nitrate, and calcium nitrate are the key components in the rooting medium. Also, a reduction in potassium nitrate to half in combination with the full amount of ammonium nitrate in MS and calcium nitrate in QL^33^ would significantly increase the rooting.

Sensitivity analysis indicated that potassium and ammonium have the maximum impact on rooting, respectively, and also indicated that ammonium nitrate has little effect at the rooting stage. The nutrient concentrations in the media would affect the rooting. Also, a number of researchers have suggested that reducing the media nutrients by half would improve the rooting^34^. The desirable effects of reducing the organic elements in rooting medium through nitrogen reduction are justified in literature^35^ which is in keeping with our findings. Nutrient reduction to half provides a satisfactory condition for root growth by reducing the vegetative growth, on the other hand, due to the reduced concentrations of nutrients, the osmotic pressure would decrease, and subsequently rooting would facilitate^36^. Our results are in contrast with those who expressed that the reduction of all macro elements by half in QL, MS and WPM^37^ would improve the rooting of the *Prunus* genus^8^. The results of the present study showed that reducing potassium nitrate and ammonium nitrate have positive effects on rooting (Tables 4 and 6). The important role of potassium and ammonium (Table 4) on rooting in this study might be due to the adjustment of pH and osmotic potential in the medium^38,39^, which is in agreement with the studies that expressed the pH as a regulating agent on absorbing the nutrients^40^. It can be inferred that potassium by adjusting the osmotic potential and ammonium by reducing the pH in the media would improve the rooting. Successful rooting is a crucial step for *in vitro* micropropagation of plants and could be a problem in some plant species micropropagation and also could limit the acclimatization of plantlets.

The results from optimizing Fe-EDDHA and Thiamine concentrations indicate that the maximum rooting obtained from modified and optimized LS medium and by changing the medium elements in all three concentrations of 100, 150 and 200 mg L^−1^ Fe-EDDHA along with 1.6 mg L^−1^ Thiamine, 100% rooting was obtained. On the other side, in a concentration of 150 mg L^−1^ Fe-EDDHA along with 2.8 mg L^−1^ thiamine also 100% rooting acquired. These results indicate that changing the medium elements would influence the efficiency of other medium components such as vitamins. The efficiency of ANN-GA to optimize the *in vitro* rooting and acclimatization of grape has been reported^26,32^ which confirms the competence of ANN-GA as a tool for optimizing *in vitro* rooting. Similar to our results, IBA has been suggested as a suitable hormone for *in vitro* rooting of *Prunus* rootstocks^9^. This is because that IBA is more stable and less sensitive to auxin degrading enzymes, and would slowly be metabolized by the peroxidase enzyme^41,42^.

In a rooting study on GF677 the concentration of 0.5 mg L^−1^ IBA caused the maximum rooting^43^. Inasmuch as the positive effect of dark has been proved^44^, the proliferated micro-shoots were kept in the dark for a week and then were transferred to the lighting conditions. Photoperiod is another factor that can affect the rooting process, the positive effects of low darkness periods on *in vitro* rooting have been reported^45^. Photoreceptors are the most important agents that cause the positive effect of dark on rooting. Phytochrome is a well-characterized plant photoreceptor, which controls the development of plants such as apical dominance and *in vitro* rooting^9^. In some species *in vitro* rooting only occurred in the presence of light. Similar to our findings in some other species the light plays as a rooting inhibitor and short-term maintenance in the dark would increase the rooting^19,46^. Dark environments inhibit the activity of phytochrome and in this condition the presence of auxins such as IBA could induce the cell division and subsequently increase the R%^47^. Removing light would protect auxin from degradation and reduce^48^ and reduces the activity of peroxidase which is an auxin degrading enzyme^49^.The positive effects of dark conditions in the present study could be derived from aforesaid reasons. According to our results, higher concentrations of IBA in rooting stage enhanced the callus formation which subsequently weakened or killed a high percentage of plants in the acclimatization stage. Callus formation at the end of the micro-shoots, impairs the vascular connection between roots and micro-shoots and prevents the absorption of water and nutrients.

In the present study the Fe-EDDHA and Thiamine compounds were used to increase the quality and R% and also to decrease the callus formation rate as well. Plantlets growth and survival during the acclimatization and reducing the losses at this stage is of great economic value. Literature indicates that, the Fe-EDTA chelate complex is not steady and causes the 45% loss of its initial Fe concentration at pH 5.8^19^. Also, decrease in Fe availability, precipitation, and production of toxic compounds have been reported in the application of Fe-EDTA as an iron source^18^. Due to the positive effects of Fe-EDDHA and Thiamine on rooting reported from several studies, these two compounds were added to LS media for increasing the quality and quantity of rooting, which produced the maximum R%.

The positive effects of iron could be attributed to its vital roles in many metabolic pathways such as cytochromes, DNA biosynthesis, hormones, lipids, detoxification of reactive oxygen species (ROS), and nitrogen assimilation; which needs sufficient amount of iron^50^. Iron deficiency leads to morphological and physiological changes in root meristem especially the root tip meristems. Some of these changes include the increased cell division, root elongation inhibition, increased root diameter, and formation of hairy roots^51^. By replacing the Fe-EDDHA with Fe-EDTA the above problems can be overcome since this compound is more stable and is easily accessible for plant uptake. Thiamine has many positive effects on numerous physiological processes such as glycolysis, the pentose phosphate pathway, and the synthesis of nucleic acids. Also, in addition to its nutritional role thiamine has been found to act as secondary signaling messengers and have been reported to induce systemic acquired resistance in plants against infections caused by various pathogens^52^.

## Conclusion

In conclusion, the results of the present study demonstrate that the type of culture media, Fe-EDDHA and Thiamine are effective factors for *in vitro* rooting of G×N15 rootstocks which have been optimized here step-by-step. The most commercial culture media for *in vitro* rooting of G×N15 obtained from the LS medium optimized by ANN-GA contained 1 mg L^−1^ IBA, 100, 150, or 200 mg L^−1^ Fe-EDDHA and 1.6 mg L^−1^ Thiamine which has been optimized through six experiments. Also, the results obtained here similar to those reported by^23,30,31^ indicate that the ANN-GA is an efficient method to formulate a new culture medium. Difference in nutritional requirements, Fe-EDDHA and Thiamine in G×N15 rootstock in comparison with the other varieties of the *Prunus* genus might be due to the different nutritional needs of various cultivars and rootstocks, internal hormone levels and physiological conditions of micro-shoots. Here, we optimized a special rooting culture medium by using ANN-GA method for the first time and increased the success of rooting G×N15 rootstock *in vitro* plantlets. The efficiency of this method for G×N15 was practically confirmed by achieving 100% rooting using the ANN-GA optimized medium. Further works are recommended to evaluate the ANN-GA method proficiency on micropropagation process of other plant species and on other components of culture medium like micro nutrients to increase the plant tissue culture technique commercial revenue.

## Methods

An overview of the various steps used in the present study to achieve an optimized *in vitro* protocol for rooting of G×N15 *prunus* rootstock has been shown in Fig. 1.

### *In vitro* culture establishment

After disinfection, the explants were cultured on a medium for shoot induction, consisting MS medium^53^, 30 g L^−1^ sucrose, 0.25 mg L^−1^ BAP, 0.05 mg L^−1^ IBA, and 7 g L^−1^ agar. The pH of applied media was adjusted to 5.8 with 0.1 M NaOH before autoclaving at 121 °C under a pressure of 1.2 kg cm−^2^ for 15 min. The explants were maintained in a growth room with light intensity of 35–40 umol m^−1^ s^−1^, 25 ± 1 °C and 16/8 h photoperiod for four weeks. Shoots that originated from the explants were subcultured after 30 days. The new micro-shoot was transferred to Yadollahi, Arab and Shojaeiyan (YAS)^20^ medium supplemented with 1 mg L^−1^ BAP and 0.1 mg L^−1^ IBA. Before direct transferring the micro-shoots to the experimental media, proliferated explants pre-subcultured four times on free hormone YAS medium for 15–20 days in order to sterility screening and fifth subculture explants were used for rooting experiments. In all rooting experiments, in order to achieve the best medium and hormonal composition of *in vitro* rooting of G×N15 rootstocks, after culturing on different media, the explants (elongated micro-shoots) were kept in the dark for the first week of culture and then transferred to 8 hours of light and 16 hours of darkness condition.

### Media preparation for *in vitro* rooting

Different culture media were applied: MS^53^, EM^9^ (Specific media), LS^54^ (Table 8),½MS modified LS predicted-optimized according to ANN-GA, and modified LS (second experiment) (Tables 6 and 9). The media were supplemented with 20 g L^−1^ sucrose and adjusted to pH 5.7–5.8 before the addition of the gelling agent (7.0 g L^−1^ ${\rm{agar}}$). After autoclaving (121 °C, 1.2 kg cm^−2^, 30 min), the media were cooled to 65 °C in a water bath, and then the medium was distributed into glass baby food jars (250 ml) each containing 50 ml of medium.

### Interaction of culture media and IBA concentrations on rooting

For the first experiment, 20 media (Table 1), differing in nutrient formulation (MS, EM, ½ MS and LS) and various concentrations of IBA as an auxin resource were evaluated. The IBA concentrations were variable, ranging from 0–2 mg L^−1^.

### Interaction of Fe-EDDHA and thiamin vitamin on rooting

For the second experiment, 12 media (Table 2), micro-shoots were cultured in LS medium supplemented with different concentrations of thiamine (0.4, 1.6, 2.8 and 4 mg L^−1^) and Fe-EDDHA at various concentrations (100, 150 and 200 mg L^−1^). All media were supplemented with 1 mg L^−1^ IBA as auxin source.

### Modeling and optimization of the obtained culture medium nutrients composition using ANN-GA

#### Preparation of LS culture media with modified macro elements concentrations

To investigate the effects of macro elements, Potassium Nitrate (KNO_3_), Ammonium Nitrate (NH_4_NO_3_), Calcium Nitrate (Ca(NO_3_)_2_) and Calcium Chloride (CaCl_2_), on optimizing the rooting of G×N15 rootstocks, 36 culture media were used (Tables 6 and 9). In order to prepare the different macro elements culture media, the LS culture medium was changed as follows: In this experiment the values of 1, 0.75 and 0.5 fold of KNO_3_ (950, 1425, 1900 mg L^−1^), values of 1, 0.75 and 0.5 fold of NH_4_NO_3_ (825, 1238, 1650 mg L^−1^) and values of 1 and 0.5 fold of Ca(NO_3_)_2_ (278 and 556 mg L^−1^) or values of 1 and 0.5 fold of CaCl_2_ (220 and 440 mg L^−1^) were combined with each other as factorial experiments based on a completely randomized design with 6 replicates each including four explants. Micro elements and vitamins used in all culture media were the same of LS medium and the amount of 1 mg L^−1^ IBA hormone was used in all culture media as well.

**Table 6:** Ion concentrations of the different culture media used for *in vitro* rooting of G×N15 rootstock.

Media	Ion concentrations (mM)					Root				
	NO_3_^−^	NH_4_^+^	K^+^	Ca^2+^	Cl^−^	RN	RL	R%	FW	DW
1	44.12	20.62	18.79	2.35	0	8.17 ± 0.31	7.08 ± 0.05	0.43 ± 0.02	0.43 ± 0.01	0.07 ± 0.004
2	41.78	20.62	18.79	1.18	0	7.33 ± 0.42	7.42 ± 0.08	0.41 ± 0.01	0.48 ± 0.01	0.09 ± 0.001
3	38.97	15.47	18.79	2.35	0	7.16 ± 0.31	7.03 ± 0.08	0.41 ± 0.02	0.40 ± 0.01	0.06 ± 0.006
4	36.63	15.47	18.79	1.18	0	6.33 ± 0.33	7.55 ± 0.04	0.36 ± 0.01	0.46 ± 0.01	0.09 ± 0.006
5	33.81	10.31	18.79	2.35	0	5.67 ± 0.33	7.11 ± 0.04	0.36 ± 0.02	0.36 ± 0.005	0.04 ± 0.004
6	31.47	10.31	18.79	1.18	0	5.50 ± 0.34	7.45 ± 0.04	0.37 ± 0.01	0.42 ± 0.007	0.07 ± 0.004
7	39.42	20.62	14.09	2.35	0	10.16 ± 0.31	6.75 ± 0.06	0.71 ± 0.01	0.51 ± 0.005	0.10 ± 0.003
8	37.08	20.62	14.09	1.18	0	10.16 ± 0.60	7.00 ± 0.04	0.69 ± 0.01	0.56 ± 0.007	0.13 ± 0.004
9	34.27	15.47	14.09	2.35	0	10.00 ± 0.58	6.80 ± 0.04	0.65 ± 0.01	0.58 ± 0.006	0.15 ± 0.008
10	31.93	15.47	14.09	1.18	0	8.33 ± 0.31	7.20 ± 0.05	0.61 ± 0.01	0.51 ± 0.01	0.10 ± 0.006
11	29.11	10.31	14.09	2.35	0	8.16 ± 0.31	7.02 ± 0.02	0.52 ± 0.02	0.47 ± 0.008	0.08 ± 0.004
12	26.77	10.31	14.09	1.18	0	7.50 ± 0.43	7.33 ± 0.05	0.51 ± 0.01	0.53 ± 0.006	0.12 ± 0.005
13	34.73	20.62	9.40	2.35	0	15.00 ± 0.36	6.82 ± 0.05	0.97 ± 0.01	0.69 ± 0.009	0.18 ± 0.013
14	32.39	20.62	9.40	1.18	0	13.33 ± 0.49	6.50 ± 0.04	0.95 ± 0.01	0.74 ± 0.006	0.23 ± 0.008
15	29.58	15.47	9.40	2.35	0	11.00 ± 0.36	6.98 ± 0.03	0.90 ± 0.01	0.67 ± 0.004	0.17 ± 0.010
16	27.24	15.47	9.40	1.18	0	9.33 ± 0.33	6.63 ± 0.05	0.85 ± 0.01	0.63 ± 0.006	0.15 ± 0.009
17	24.42	10.31	9.40	2.35	0	9.00 ± 0.26	6.71 ± 0.02	0.80 ± 0.01	0.58 ± 0.008	0.13 ± 0.007
18	22.08	10.31	9.40	1.18	0	8.00 ± 0.26	6.41 ± 0.03	0.75 ± 0.01	0.53 ± 0.007	0.11 ± 0.006
19	39.41	20.62	18.79	3.00	3.00	5.16 ± 0.31	7.61 ± 0.05	0.34 ± 0.01	0.34 ± 0.027	0.05 ± 0.007
20	39.41	20.62	18.79	1.50	1.50	6.67 ± 0.33	7.20 ± 0.06	0.38 ± 0.01	0.37 ± 0.025	0.07 ± 0.008
21	34.26	15.47	18.79	3.00	3.00	6.17 ± 0.16	7.37 ± 0.04	0.32 ± 0.01	0.39 ± 0.008	0.06 ± 0.005
22	34.26	15.47	18.79	1.50	1.50	5.50 ± 0.22	7.48 ± 0.03	0.36 ± 0.008	0.34 ± 0.016	0.05 ± 0.008
23	29.10	10.31	18.79	3.00	3.00	5.17 ± 0.17	7.52 ± 0.03	0.32 ± 0.010	0.35 ± 0.013	0.06 ± 0.008
24	29.10	10.31	18.79	1.50	1.50	4.67 ± 0.42	7.75 ± 0.07	0.28 ± 0.011	0.33 ± 0.013	0.04 ± 0.004
25	34.71	20.62	14.09	3.00	3.00	9.67 ± 0.21	6.80 ± 0.06	0.59 ± 0.015	0.54 ± 0.014	0.12 ± 0.009
26	34.71	20.62	14.09	1.50	1.50	10.33 ± 0.33	6.63 ± 0.09	0.62 ± 0.011	0.59 ± 0.012	0.15 ± 0.011
27	29.56	15.47	14.09	3.00	3.00	7.50 ± 0.22	7.10 ± 0.06	0.52 ± 0.010	0.50 ± 0.013	0.10 ± 0.004
28	29.56	15.47	14.09	1.50	1.50	7.67 ± 0.21	7.00 ± 0.08	0.53 ± 0.011	0.56 ± 0.014	0.13 ± 0.008
29	24.40	10.31	14.09	3.00	3.00	6.83 ± 0.17	7.17 ± 0.11	0.46 ± 0.020	0.44 ± 0.018	0.08 ± 0.006
30	24.40	10.31	14.09	1.50	1.50	7.33 ± 0.21	6.98 ± 0.10	0.48 ± 0.010	0.48 ± 0.011	0.10 ± 0.007
31	30.02	20.62	9.40	3.00	3.00	10.83 ± 0.31	6.48 ± 0.05	0.83 ± 0.016	0.61 ± 0.008	0.17 ± 0.005
32	30.02	20.62	9.40	1.50	1.50	12.17 ± 0.48	6.28 ± 0.04	0.87 ± 0.011	0.67 ± 0.009	0.22 ± 0.009
33	24.87	15.47	9.40	3.00	3.00	10.00 ± 0.36	6.70 ± 0.08	0.77 ± 0.011	0.55 ± 0.007	0.12 ± 0.004
34	24.87	15.47	9.40	1.50	1.50	9.83 ± 0.17	6.55 ± 0.07	0.82 ± 0.010	0.59 ± 0.009	0.15 ± 0.009
35	19.71	10.31	9.40	3.00	3.00	8.50 ± 0.22	7.00 ± 0.08	0.67 ± 0.021	0.48 ± 0.008	0.10 ± 0.004
36	19.71	10.31	9.40	1.50	1.50	9.00 ± 0.36	6.85 ± 0.09	0.74 ± 0.015	0.54 ± 0.014	0.13 ± 0.007

**Table 7:** Effect of different concentration of Fe-EDDHA and Thiamine on root number (RN), root length (RL), root percentage (R%), root fresh weight (FW) and root dry weight (DW) of G×N15 *prunus* rootstock plantlets during *in vitro* rooting.

Effects		RN	RL	R%	FW	DW
**Thiamine × Fe-EDDHA (mg L** ^−**1**^ **)**						
0.4	100	8.8 ± 0.37 d	9.24 ± 0.10 de	0.80 ± 0.05 b	0.38 ± 0.004 de	0.08 ± 0.004 de
	150	11.6 ± 0.51 b	10.28 ± 0.17 bcd	1.00 ± 0.00 a	0.45 ± 0.011 b	0.09 ± 0.003 d
	200	8.0 ± 0.05 d	7.02 ± 0.18 gh	0.75 ± 0.00 b	0.35 ± 0.003 e	0.07 ± 0.001 ef
1.6	100	12.0 ± 0.32 b	11.28 ± 0.19 b	1.00 ± 0.00 a	0.44 ± 0.009 bc	0.12 ± 0.003 bc
	150	16.0 ± 0.71 a	15.66 ± 0.56 a	1.00 ± 0.00 a	0.57 ± 0.020 a	0.16 ± 0.011 a
	200	10.0 ± 0.55 bcd	9.28 ± 0.15 de	1.00 ± 0.00 a	0.44 ± 0.008 bc	0.12 ± 0.005 bc
2.8	100	9.4 ± 0.40 cd	9.86 ± 0.27 cd	0.80 ± 0.05 b	0.40 ± 0.006 cd	0.10 ± 0.007 cd
	150	11.0 ± 0.45 bc	10.68 ± 0.18 bc	1.00 ± 0.00 a	0.44 ± 0.006 bc	0.12 ± 0.002 b
	200	8.2 ± 0.37 d	8.20 ± 0.15 ef	0.90 ± 0.06 ab	0.36 ± 0.001 de	0.06 ± 0.003 efg
4	100	3.6 ± 0.24 e	6.26 ± 0.08 h	0.45 ± 0.05 c	0.18 ± 0.007 g	0.04 ± 0.002 gh
	150	4.0 ± 0.45 e	8.02 ± 0.25 fg	0.35 ± 0.06 cd	0.23 ± 0.007 f	0.05 ± 0.001 fgh
	200	3.4 ± 0.24 e	6.26 ± 0.13 h	0.25 ± 0.00 d	0.17 ± 0.001 g	0.04 ± 0.001 h
**P-Value**						
Thiamine × Fe-EDDHA	<0.001	<0.001	<0.001	<0.001	<0.001	<0.001

Values in each column represent means ± SE. Different letters within columns indicate significant differences.

**Table 8:** MS^53^, EM^9^ and LS^54^ culture media compositions.

Micro Elements	MS (mg L^−1^)	EM (mg L^−1^)	LS (mg L^−1^)
CoCl_2_.6H_2_O	0.025	0.025	0.025
CuSO_4_.5H_2_O	0.025	0.025	0.025
FeNaEDTA	36.70	36.70	36.70
H_3_BO_3_	6.2	6.20	6.2
KI	0.83	0.83	0.83
MnSO_4_.7H_2_O	22.3	22.3	22.3
Na_2_MoO_4_.2H_2_O	0.25	0.25	0.25
ZnSO_4_.7H_2_O	8.60	8.60	8.60
**Macro Elements**			
CaCl_2_.2H_2_O	440	—	440
Ca(NO_3_)_2_.4H_2_O	—	801.77	—
KH_2_PO_4_	170.0	116.08	170.0
KNO_3_	1900	1168.8	1900
MgSO_4_.7H_2_O	370	100.38	370
MgCl_2_.6H_2_O	—	531.94	—
NH_4_NO_3_	1650	2502.5	1650
**Vitamins**			
myo-Inositol	100	100	100
Thiamine HCl	0.1	0.40	0.1
Pyridoxine HCl	0.5	0.5	—
Nicotinic Acid	0.5	0.5	—
Glycine	2	2	—

**Table 9:** Mineral composition of the different culture media for *in vitro* rooting of G×N15 rootstock.

Code	Macronutrients (mg L^−1^)			
	**KNO**_**3**_	**NH**_**4**_ **NO**_**3**_	**Ca(NO**_**3**_ **)**_**2**_	**CaCl**_**2**_
1	1900	1650	556	0
2	1900	1650	280	0
3	1900	1238	556	0
4	1900	1238	280	0
5	1900	825	556	0
6	1900	825	280	0
7	1425	1650	556	0
8	1425	1650	280	0
9	1425	1238	556	0
10	1425	1238	280	0
11	1425	825	556	0
12	1425	825	280	0
13	950	1650	556	0
14	950	1650	280	0
15	950	1238	556	0
16	950	1238	280	0
17	950	825	556	0
18	950	825	280	0
19	1900	1650	0	440
20	1900	1650	0	220
21	1900	1238	0	440
22	1900	1238	0	220
23	1900	825	0	440
24	1900	825	0	220
25	1425	1650	0	440
26	1425	1650	0	220
27	1425	1238	0	440
28	1425	1238	0	220
29	1425	825	0	440
30	1425	825	0	220
31	950	1650	0	440
32	950	1650	0	220
33	950	1238	0	440
34	950	1238	0	220
35	950	825	0	440
36	950	825	0	220

**Table 10:** Effect of different media on root number (RN), root length (RL), rooting percentage (R%), fresh weight (FW) and dry weight (DW).

Media	RN	RL	R%	FW	DW
MS	6.0 ± 0.31 d	5.44 ± 0.05 d	0.32 ± 0.01 c	0.19 ± 0.008 d	0.03 ± 0.003 c
EM	4.6 ± 0.25 d	4.28 ± 0.09 e	0.37 ± 0.01 c	0.10 ± 0.007 e	0.014 ± 0.002 c
Modified LS	15.4 ± 0.51 a	8.10 ± 0.07 a	0.86 ± 0.02 a	0.70 ± 0.015 a	0.16 ± 0.006 a
½ MS	8.8 ± 0.37 c	6.33 ± 0.05 c	0.73 ± 0.01 b	0.43 ± 0.013 c	0.10 ± 0.005 b
LS	13.6 ± 0.40 b	7.42 ± 0.06 b	0.78 ± 0.02 b	0.59 ± 0.015 b	0.12 ± 0.005 b
P-Value	<0.001	<0.001	<0.001	<0.001	<0.001

Values in each column represent means ± SE. Different letters within columns indicate significant differences.

### ANN-GA modeling

#### Developing ANN models

Five data matrices including 36 mineral composition treatments with different concentrations of ions NO_3_^−^, NH_4_^+^, K^+^, Ca^2+^ and Cl^−^ (Table 6) were developed for each of the five rooting measured parameters including RN, RL, R%, FW and DW to perform ANN modeling.

Developing an ANN is according to the operations summation in each neuron that constitute the system. A vector *X*_*i*_ = (X1, X2, … X_n_) is used for entering the information into the system. A mathematical function is used for processing the information of the input vector which transfers them to the first intermediate layer. The commitment of propagation function is adding all the input data and generating one response (Equation 1). where, N is the number of neurons in the first ANN layer, denominated input layer, *w*_*ni*_ is the weight (indicating the importance of the connection) between neurons of the input layer (n) and neurons of the middle layer (i), and b is the biases related to the neurons in the middle layer (Equation 1).

1 $${S}_{i}={\sum }_{n-1}^{N}{w}_{ni}{x}_{n}+{b}_{i}$$

The obtained values by the propagation function are applied by another mathematical function, named activation function (Equation 2), to make an output value (y) as a function of the internal state^55^ and is more than a threshold value^33^. Different activation functions can be used but in this work, Tansig (hyperbolic tangent sigmoid) (Equation 2) and purelin (linear) functions were applied as the transfer functions for hidden and output layers, respectively.

2 $$y=\frac{2}{1+\exp (-2\,\ast \,n)}-1$$

All entered information in the ANN are propagated to the output layer, where an output value is made (y_0_) which is compared with the observed value (d_0_), and the mean squared error (MSE) produced by the ANN (Eq. 3) can be estimated.

3 $$MSE=1/2{\sum }_{i=1}^{n}{({d}_{0}-{y}_{0})}^{2}$$

To train the network, a Levenberg-Marquardt algorithm for back-propagation with a gradient descent and momentum weight and bias learning function was applied^56^. The MSE leveling 0.01 was applied as performance function and training was ended after 800 epochs or iterations of the network. Different levels of five input variables including NO_3_^−^, NH_4_^+^, Ca^2+^, K^+^ and Cl^-^ were applied as units in the input layer of the ANN model. Five models were developed separately for RN, RL, R%, FW and DW with the topology of 5–8–1. For training and testing of the network, 130 and 86 data lines were used. The data lines in train and test groups were randomly chosen before establishing the training model. Prior to training, the input and output data were normalized in the range of −1 to 1 to simplify the problem for the network, to reach fast convergence minimum mean square error, and to guarantee that the fall of targets (output data) into the certain range of the new feed forward network could be reconstructed^56–58^. The fitness of the ANN-model was assessed by R^2^, root mean square error (RMSE) and mean bias error (MBE) as follows:

4 $${R}^{2}=1-\frac{{\sum }_{i=1}^{n}{(M-O)}^{2}}{{\sum }^{}(O-\bar{O})}$$

5 $$RMSE=\sqrt{(\sum _{i=1}^{n}{(M-O)}^{2})/n}$$

6 $$MBE=1/n{\sum }_{i=1}^{n}|M-O|$$

where n is the number of data, O is the value of actual datasets, and M is value of predicted datasets. The ranges are 0 ≤ R^2^ ≤ 1, 0 ≤ RMSE ≤  + ∞ and −1 ≤ MBE ≤ +1. R^2^ values closer to 1 and RMSE values closer to 0 indicate better fit. The MBE value represents a positive or negative calculation error which indicate that the predicted values are more or less than observational values.

#### Optimization of input variables for the developed ANN models using GA

**Figure 7:**
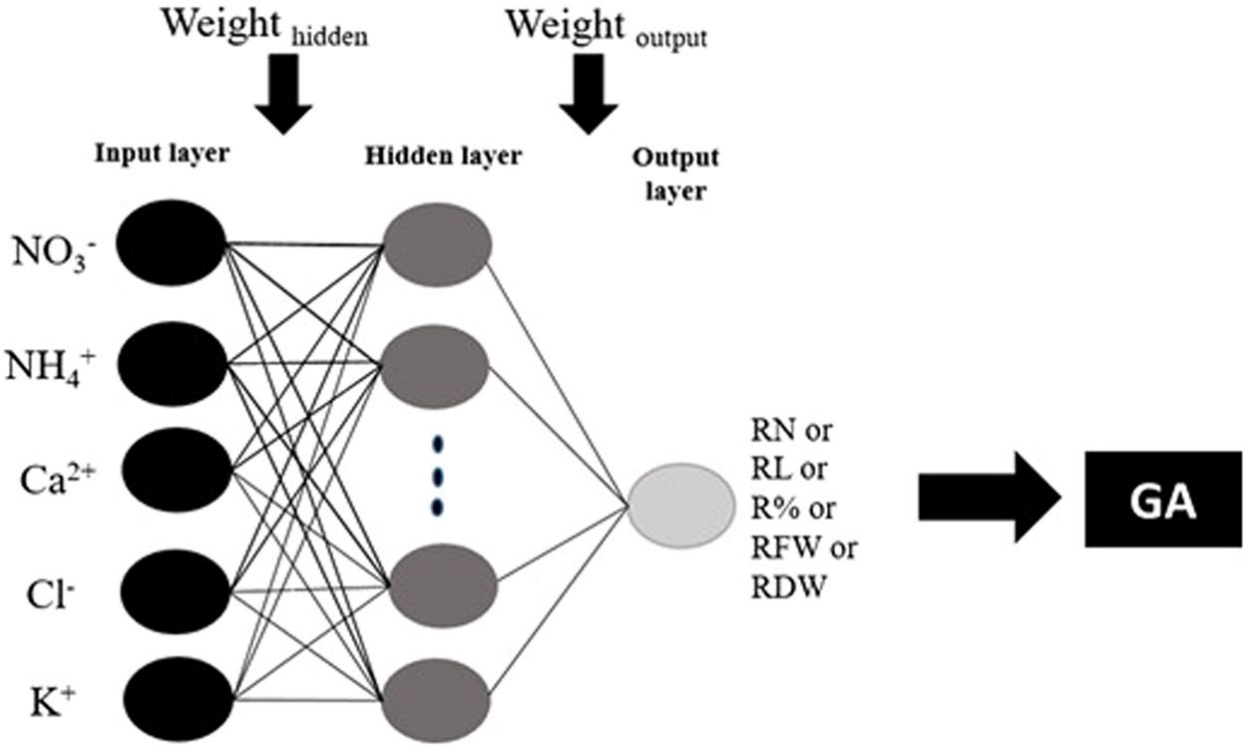
Schematic diagram of an artificial neural network (ANN) model with 5 input neurons, 8 neurons in the intermediate layer and one output neuron (including one of RN: root number, RL: root length, R%: root percentage, root fresh weight: FW or root dry weight: DW) with the topology of 5–8–1. The connections between nodes are related to weights and biases.

**Figure 8:**
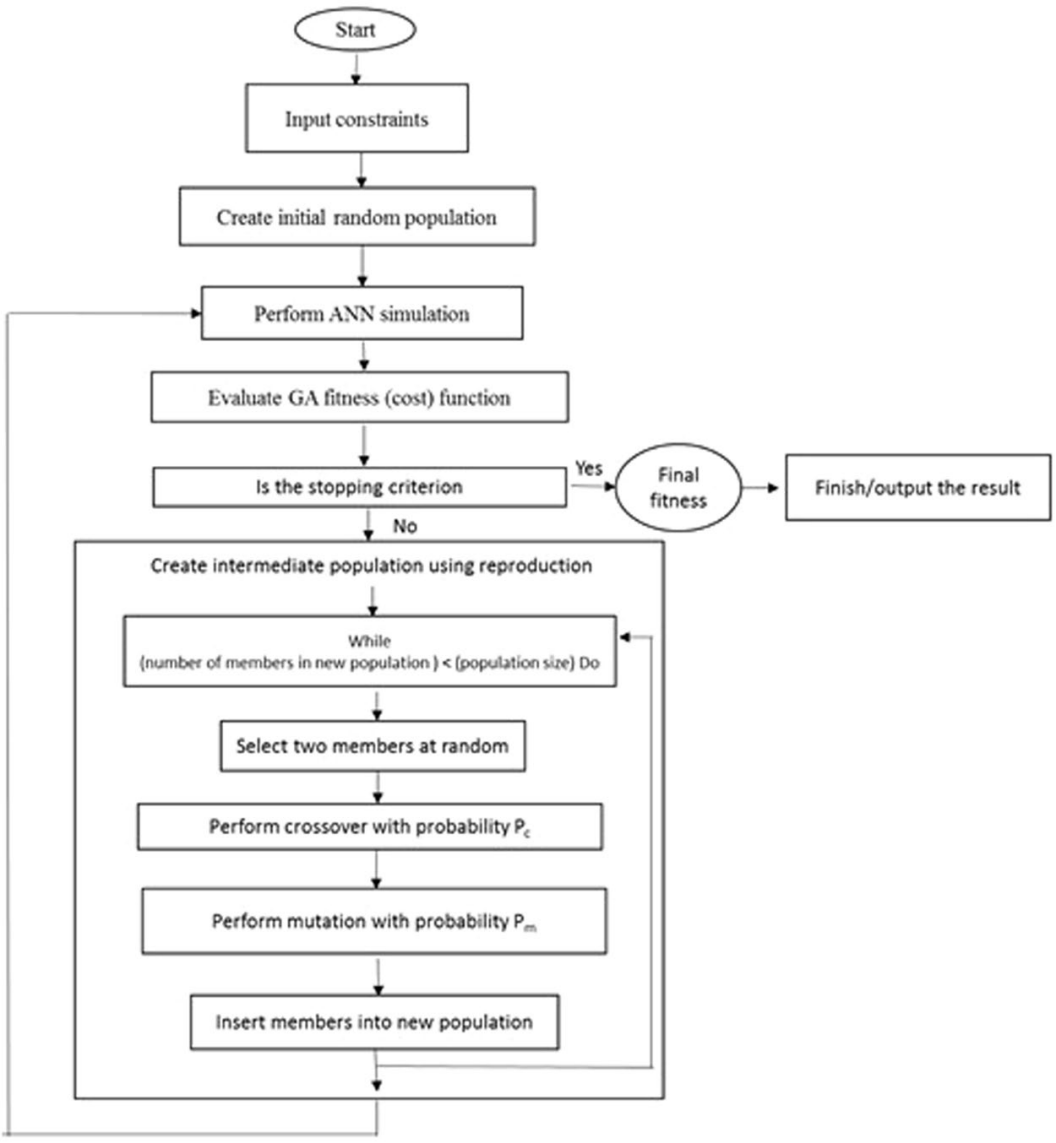
Schematic diagram showing the relationship between ANN and GA to achieve an optimized model (adopted from^20^).

To determine the optimal values of input variables (KNO_3_, NH_4_NO_3_, Ca(NO_3_)_2_, and CaCl_2_) and maximize the RN, RL, R%, FW and DW, the prepared ANN models were exposed to a further process using GA, after the training process. Accordingly, the ANN models were applied as the fitness function for GA (Figs 7 and 8). For selecting the elite populations for crossover a roulette wheel selection method was applied. To attain the best fitness, the initial population of 50, generation number of 500, mutation rate of 0.1, and crossover rate of 0.85 were set^59^. This generational process was repeated until the number of generations was reached. To identify the important input variable in the model, the constructed ANN models were subjected to the sensitivity analysis. This analysis indicates which KNO_3_, NH_4_NO_3_, Ca(NO_3_)_2_, and CaCl_2_ concentration is more important to acquire the optimal number of root, root length, root percentage, and fresh and DW in G×N 15 rootstock micro-shoots.

### Sensitivity analyses

The sensitivity of number of root, root length, root percentage, and fresh and DW against the investigating media nutrients was determined using the criteria^60,61^ as follows:

- The variable sensitivity error (VSE) value: demonstrates the performance of the developed ANN model if that variable is unavailable,
- The value of variable sensitivity ratio (VSR): is the relative ratio between the VSE and the error of the ANN model when all variables are available. A variable which is more important has a higher VSR value. Therefore, according to the obtained VSR value, the input variables may be ranked in the order of importance.

### Validation experiments

In this experiment different culture media were employed: MS medium^53^, ½ MS, EM (Specific media), LS, and modified LS medium containing predicted-optimized mineral nutrient based on ANN-GA. The media were supplemented with 1 mg/ l IBA, 20 g /l sucrose, 100 mg L^−1^ myo-inositol (Sigma). The pH of all media was adjusted to pH 5.7–5.8 prior to the addition of a gelling agent (7.0 g L^−1^ agar).

### Optimizing thiamin and Fe-EDDHA for the ANN-GA optimized LS medium

In this experiment, the micro-shoots were cultured in modified LS medium supplemented with different concentrations of thiamine (0.4, 1.6, 2.8 and 4 mg L^−1^) and Fe-EDDHA at various concentrations (100, 150 and 200 mg L^−1^). All media were supplemented with 1 mg L^−1^ IBA as Auxin resource.

### Experiments design and data collection

All experiments were conducted by a factorial experiment based on a completely randomized design with five (first, fourth, fifth and sixth experiment) to six (second and third experiment) replicates and each replication included 4 explants. At the end of *in vitro* rooting stage, five parameters (outputs) were recorded to analyze the effects of the variables (inputs) on rooting: (1) total root produced (number of new roots per explant: 1, 2, 3, …); (2) Length of roots; (3) R%; (4) FW and (5) DW. Commercially available software, Matlab® R2010a^21^, was used to write the mathematical codes for developing and evaluating the ANN model. The developed program is actually a modified source code of an ANN algorithm which was previously applied by Ahmadi and Golian^57^. In the sixth experiment to evaluate the ANN-GA efficiency in the prediction and optimization of the new medium, MS, ½ MS, EM and LS basal salts and new formulated medium (Modified LS) were compared. Sixth experiment data were subjected to a one-way analysis of variance. Statistical significance was determined by analysis of variance and significance (P ≤ 0.05) differences between mean values were estimated using LSD test. SAS version 9.1 was used for statistical analyses and a value of P < 0.05 was considered significant.

The mathematical code for developing and evaluating the ANN-GA model was written by Matlab R2010a^21^ software. In fact, the developed program is a modified source code of an ANN algorithm which was previously applied by^57^.
